# Mechanisms of Sensitivity and Resistance of Primary Effusion Lymphoma to Dimethyl Fumarate (DMF)

**DOI:** 10.3390/ijms23126773

**Published:** 2022-06-17

**Authors:** Roberta Gonnella, Roberta Zarrella, Roberta Santarelli, Concetta Anna Germano, Maria Saveria Gilardini Montani, Mara Cirone

**Affiliations:** Department of Experimental Medicine, Sapienza University of Rome, Viale Regina Elena 324, 00161 Rome, Italy; roberta.gonnella@uniroma1.it (R.G.); zarrella.1907329@studenti.uniroma1.it (R.Z.); roberta.santarelli@uniroma1.it (R.S.); concettaanna.germano@uniroma1.it (C.A.G.); mariasaveria.gilardinimontani@uniroma1.it (M.S.G.M.)

**Keywords:** PEL, NRF2, ROS, STAT3, inflammatory cytokines, mTOR/p-4EBP1, ERK1/2, autophagy

## Abstract

PEL is a rare B cell lymphoma associated with KSHV that mainly arises in immune-deficient individuals. The search for new drugs to treat this cancer is still ongoing given its aggressiveness and the poor response to chemotherapies. In this study, we found that DMF, a drug known for its anti-inflammatory properties which is registered for the treatment of psoriasis and relapsing–remitting MS, could be a promising therapeutic strategy against PEL. Indeed, although some mechanisms of resistance were induced, DMF activated NRF2, reduced ROS and inhibited the phosphorylation of STAT3 and the release of the pro-inflammatory and immune suppressive cytokines IL-6 and IL-10, which are known to sustain PEL survival. Interestingly, we observed that DMF displayed a stronger cytotoxic effect against fresh PEL cells in comparison to PEL cell lines, due to the activation of ERK1/2 and autophagy in the latter cells. This finding further encourages the possibility of using DMF for the treatment of PEL.

## 1. Introduction

Dimethyl fumarate (DMF; trade name: Tecfidera), a drug registered for the treatment of relapsing–remitting multiple sclerosis (MS) and psoriasis [1], has been shown to also display anti-cancer activity [2,3]. Although the underlying mechanisms have not been completely elucidated, most DMF activities seem to be related to its capacity to deplete cellular GSH (via GSH succination) and activate nuclear factor erythroid 2 (NF-E2)-related factor 2 (NRF2) through the succination and inhibition of Kelch-like ECH-associated protein 1 (KEAP1), removing the inhibitory activity on NRF2 [4]. Indeed, the activation of NRF2 and the inhibition of nuclear factor kappa-light-chain-enhancer of activated B cells (NF-κB) [5,6] underly the capacity of DMF to reduce the inflammatory process. Through NRF2 activation, DMF modulates the expression of a variety of anti-oxidant enzymes, including heme oxygenase 1 (HO-1) and NADPH dehydrogenase (quinone 1) (NQO1), thus dysregulating the intracellular radical oxygen species (ROS) level, whose fine balance is required for cancer cell survival [7]. Accordingly, we have previously shown that Apigenin (a natural compound abundantly contained in vegetables and fruits) inhibited signal transducer and activator of transcription 3 (STAT3) [8] (a pathway whose activation plays a key role in the survival of Primary Effusion Lymphoma (PEL) cells [9,10]) by reducing ROS. However, Apigenin, as well as other flavonoids, has been reported to mediate its cytotoxic effect against other cancer types by increasing ROS rather than reducing them [11], which nonetheless confirms the dependency of cancer cell survival on the appropriate amount of these molecules, which are involved in multiple biological processes [12].

PEL is a rare, aggressive B cell lymphoma carrying Kaposi’s Sarcoma Herpesvirus (KSHV) and, in most of the cases, also Epstein Barr virus (EBV) infection. The search for more effective therapies for this cancer is needed, given its poor response to conventional chemotherapies such as cyclophosphamide, doxorubicin, vincristine, and prednisone (CHOP) [13,14]. Drugs targeting STAT3 may represent a promising therapeutic option against this cancer [9,15,16], even if several other oncogenic pathways contribute to driving lymphoma cell survival, including PI3K/AKT/mTOR, NFkB, ERK1/2 and mevalonate [17,18,19,20,21]. Interestingly, STAT3 has also been reported to be among the pathways inhibited by DMF, e.g., in hepatocellular carcinoma [22]. AKT/mTOR/ERK signaling has also been shown to be inhibited by DMF in melanoma cells when used in combination with Vemurafenib [23]. Based on this collection of evidence and the anti-inflammatory activity of DMF, in this study, we investigated the possibility of treating PEL cell lines and fresh PEL cells with this drug. Indeed, besides the above-mentioned oncogenic pathways, PEL survival strongly relies on the autocrine effect of pro-inflammatory cytokines such as IL-6 [24], whose release is strictly interconnected with the activation of oncogenic pathways such as STAT3 [25,26]. Moreover, the impact of DMF on autophagy was evaluated in PEL cell lines and fresh PEL cells, given that the autophagic process, interconnected with several oncogenic pathways, may strongly influence the response to anti-cancer treatments [27].

## 2. Results

### 2.1. DMF Impairs PEL Cell Survival by Activating NRF2, Reducing Intracellular ROS and STAT3 Activation, and Counteracting the Release of IL-6 and IL-10 Cytokines

**Figure 1:**
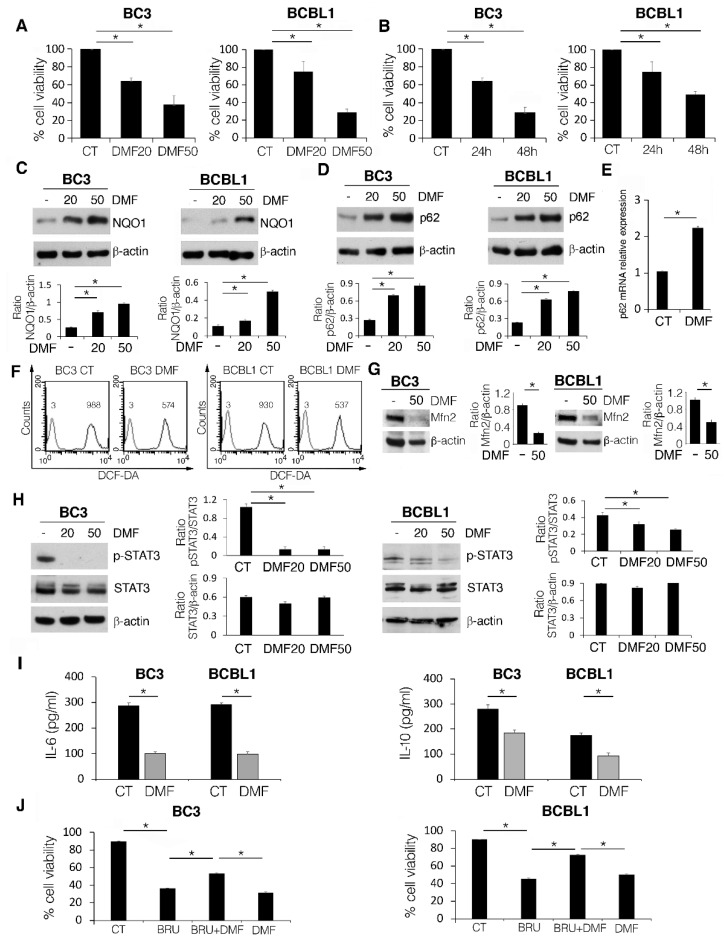
DMF cytotoxicity in PEL cell lines is mediated by NRF2 activation and reduction of intracellular ROS and IL-6 and IL-10 release. (**A**,**B**) Percentage of cell viability of BC3 and BCBL1 cells, treated or not (CT) with 20 and 50 μM DMF for 24 h, or with 20 μM for 24 and 48 h, as evaluated by Trypan blue exclusion assay. (**C**) NQO1 and (**D**) p62/SQSTM (p62) expression levels in BC3 and BCBL1 cells, treated or not (CT) with 20 and 50 µM DMF for 24 h, assessed by western blotting. β-actin was used as loading control, and one representative experiment out of three is shown. The histograms represent the mean plus SD of the densitometric analysis of the NQO1/β-actin and p62/β-actin ratios of three different experiments. (**E**) p62 mRNA level of BC3 cells, treated or not (CT) with 50 µM DMF for 24 h, was quantitated by qRT-PCR. (**F**) FACS analysis using DC-FDA staining to measure the intracellular ROS in BC3 and BCBL1 cells, treated or not (CT) with 50 µM DMF for 24 h. (**G**) Western blotting showing MFN2 or (**H**) STAT3 phosphorylation (pSTAT3) in BC3 and BCBL1 cells, treated or not (CT) with 20 and 50 µM DMF for 24 h. β-actin was used as loading control, and one representative experiment out of three is shown. The histograms represent the mean plus SD of the densitometric analysis of the pSTAT3/STAT3 and STAT3/β-actin ratios of three different experiments. (**I**) IL-6 and IL-10 release by BC3 and BCBL1 cells, treated or not (CT) with 50 µM DMF for 24 h. (**J**) Percentage of cell viability of BC3 and BCBL1 cells, co-treated with 20 µM DMF and 5 nM Brusatol (BRU) for 24 h, evaluated by Trypan blue exclusion assay. In the figure, mean ± standard deviation (SD) of three independent experiments is shown. * *p* < 0.05.

BC3 and BCBL-1 PEL cells were treated with different concentrations (20 and 50 µM) of DMF for 24 h; cell viability was then assessed by trypan blue assay. As shown in Figure 1A,B, DMF induced a dose-dependent and a time-dependent reduction of cell survival in both cell lines. DMF is known to activate NRF2 [28]; accordingly, in this study, we found that NQO1, one of the NRF2 targets, [29], was upregulated in a dose-dependent manner by this drug (Figure 1C).

We then evaluated the expression level of p62/SQSTM1, a protein which is another NRF2 target able to establish a positive feedback loop with this transcription factor [30]. We found that p62/SQSTM1 expression increased following DMF treatment either at the protein (Figure 1D) or mRNA level (Figure 1E). In correlation with the activation of the antioxidant response, ROS were reduced by DMF, as demonstrated by FACS analysis using DCF-DA staining (Figure 1F). Of note, DMF also altered the mitochondrial dynamics, which is strictly correlated with the function of these organelles, as it downregulated Mitofusin (MFN) 2 (Figure 1G), a protein which regulates mitochondrial fusion. The reduction of ROS was previously found to be a mechanism leading to a reduction of PEL cell survival following treatment with Apigenin as well as the antioxidant N-acetylcysteine (NAC), which inhibited STAT3 by reducing ROS [8]. A proper level of ROS contributes to the activation of oncogenic pathways and thus may help tumor cell proliferation [7]. In accordance with these previous findings [22], in this study, we observed that DMF reduced STAT3 tyrosine 705 phosphorylation in both PEL cell lines (Figure 1H), suggesting that this could be a molecular mechanism underlying its cytotoxic effect.

Given that STAT3 activation is strongly interconnected with the release of the pro-inflammatory and immunosuppressive cytokines such as IL-6 and IL-10, we then performed a Luminex assay to investigate the impact of DMF on their production. As shown in Figure 1I, DMF reduced both IL-6 and IL-10 cytokines, which are required for PEL cell growth, by acting in an autocrine fashion [24]. This suggests that the interruption of the feedback loop between STAT3 and these pro-survival cytokines by DMF could play an important role in the effect exerted against PEL cells. Interestingly, we found that counteracting the activation of NRF2 by using Brusatol, the cytotoxic effect of DMF decreased in both PEL cell lines (Figure 1J), suggesting that its cytotoxicity involved NRF2 activation.

### 2.2. p-4EBP1 Activation Counteracts the Cytotoxic Effect of DMF at a Low Dose

**Figure 2:**
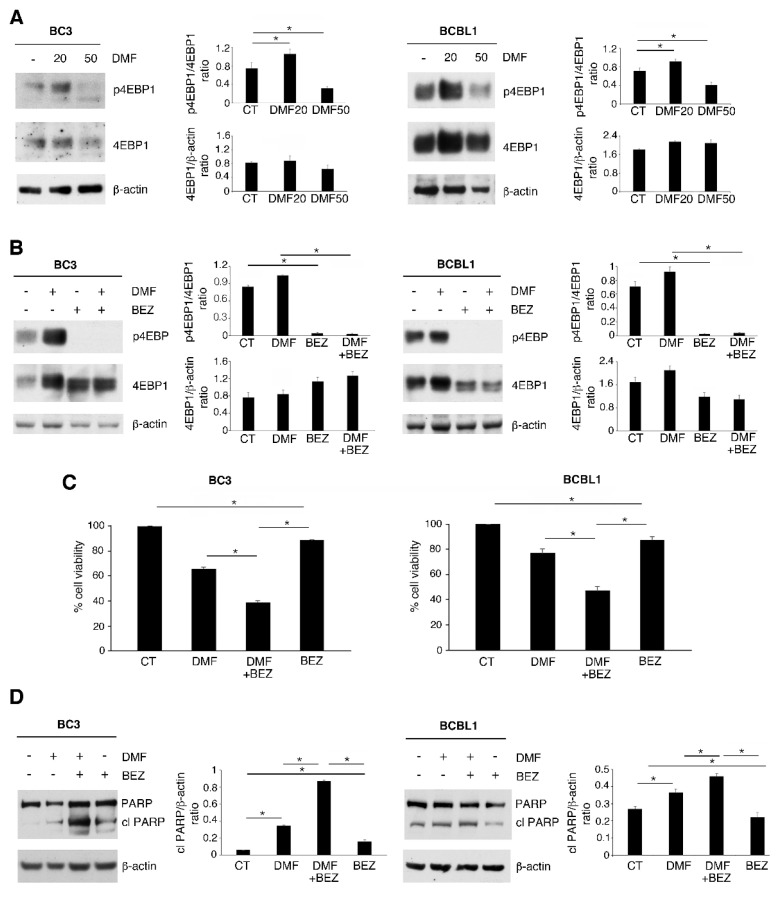
mTOR pathway activation reduces DMF cytotoxicity in PEL cell lines. Western blot analysis was used to detect 4EBP1 phosphorylation (p-4EBP1) in BC3 and BCBL1 cells, (**A**) treated or not (CT) with 20 and 50 µM DMF for 24 h and (**B**) pre-treated with 100 nM mTOR inhibitor NVP-BEZ235 (BEZ) and then exposed to 20 µM DMF. β-actin was used as loading control, and one representative experiment out of three is shown. The histograms represent the mean plus SD of the densitometric analysis of the p-4EBP1/4EBP1and 4EBP1/β-actin ratios of three different experiments. (**C**) Percentage of cell viability of BC3 and BCBL1 cells, treated or not (CT) with 20 µM DMF and 100 nM of BEZ for 24 h, was evaluated by Trypan blue exclusion assay. (**D**) PARP cleavage (cl PARP) was assessed by western blotting in BC3 and BCBL1 cells, treated or not (CT) with 20 and 50 µM DMF for 24 h. β-actin was used as loading control, and one representative experiment out of three is shown. The histograms represent the mean plus SD of the densitometric analysis of the cl PARP/β-actin ratio of three different experiments. In the figure, mean ± standard deviation (SD) of three independent experiments is shown. * *p* < 0.05.

Given that STAT3 was de-phosphorylated by DMF at both 20 and 50 µM doses, particularly in BC3 cells, we then evaluated whether the activation of pro-survival pathway(s) could counteract its cytotoxic effect when used at the lower dose of 20 µM. We investigated the phosphorylation of 4EBP1 (an mTOR target), as the activation of this pathway sustains PEL cell survival [19] and can be influenced by DMF treatment [23]. As shown in Figure 2A, at 20 µM dose, p-4EBP1 was hyperactivated by DMF in both BC3 and BCBL1 cells. To demonstrate that the activation of p-4EBP1 could represent a mechanism of resistance to DMF treatment in this setting, we used this drug at 20 µM with NVP-BEZ235, a dual mTOR inhibitor. Such a combination strongly impaired p-4EBP1 activation (Figure 2B) and potentiated the cytotoxic effect of DMF (Figure 2C) as well as PARP cleavage (Figure 2D), suggesting that mTOR activation represented a mechanism of resistance of PEL cells to the treatment with a low dose of DMF.

### 2.3. DMF Induces a Lower Cytotoxic Effect against BC3 Cell Line Compared to Fresh PEL Cells Due to ERK1/2 Activation

**Figure 3:**
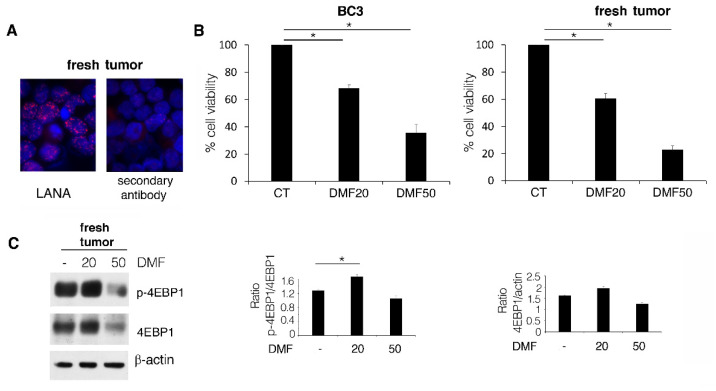

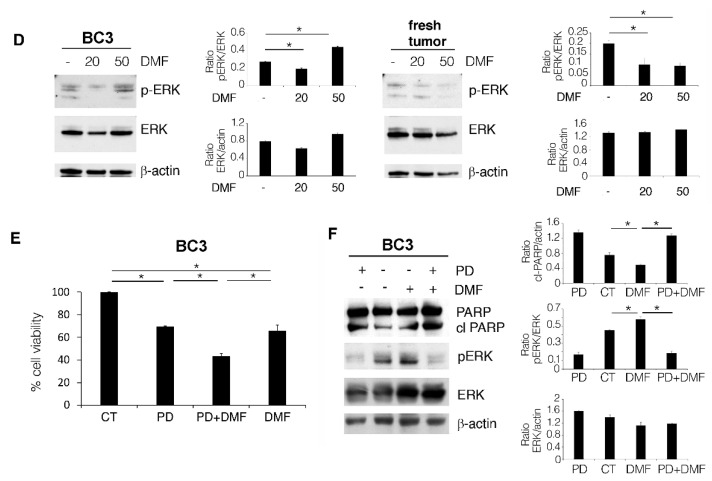
Lack of ERK1/2 activation underlies the higher cytotoxicity of DMF observed in PEL fresh tumors compared to BC3 PEL cell line. (**A**) IFA analysis to relieve LANA1 KSHV latent antigen (red) in PEL fresh tumors. DAPI (blue) was used to stain nuclei. (**B**) Percentage of cell viability of BC3 cells and PEL fresh tumors, treated or not (CT) with 20 and 50 µM DMF for 24 h, evaluated by Trypan blue exclusion assay. (**C**) PEL fresh tumors, treated or not (CT) with 20 and 50 µM DMF for 24 h, were analyzed by western blotting to assess p-4EBP1 level. β-actin was used as loading control, and one representative experiment out of three is shown. The histograms represent the mean plus SD of the densitometric analysis of the p-4EBP1/4EBP1and 4EBP1/β-actin ratios of three different experiments. (**D**) Western blotting of pERK1/2 in BC3 cells and PEL fresh tumors, treated or not (CT) with 20 and 50 µM DMF for 24 h. β-actin was used as loading control, and one representative experiment out of three is shown. The histograms represent the mean plus SD of the densitometric analysis of the pERK1/2/ERK1/2 and ERK1/2/β-actin ratios of three different experiments. (**E**) Percentage of cell viability of BC3 cells, treated with 50 µM DMF or not (CT) in the presence of 20 µM PD98059 EK1/2 inhibitor for 24 h. (**F**) PARP cleavage (cl PARP) evaluated by western blotting of BC3 cells, treated or not (CT) with 50 µM DMF for 24 h. β-actin was used as loading control, and one representative experiment out of three is shown. The histograms represent the mean plus SD of the densitometric analysis of the cl PARP/β-actin ratio of three different experiments. In the figure, mean ± standard deviation (SD) of three independent experiments is shown. * *p* < 0.05.

We then compared the cytotoxic effect exerted by DMF on PEL cell lines with that induced on fresh PEL cells grown in SCID mice. To this end, BC3 cells were engrafted i.p. and, after 35 days, the ascites was withdrawn and cells were isolated, as this lymphoma mainly grows in liquid effusions. After having checked that the cells contained in ascites were PEL cells, based on positivity for LANA1 (a latent KHSV antigen always expressed in these lymphoma cells (Figure 3A), we treated them with DMF at 20 and 50 µM. We found that fresh PEL cells were more sensitive to DMF than the BC3 cell line when the drug was used at the 50 µM dose (Figure 3B). Indeed, at the lower dose of 20 µM, DMF also induced the activation of p-4EBP1 in these cells (Figure 3C). Searching for the molecular mechanism(s) underlying the different cytotoxicity of DMF used at 50 µM against BC3 and fresh PEL cells, we observed an increase of ERK1/2 phosphorylation in the BC3 cell line, an effect that was not induced by DMF in fresh PEL cells (Figure 3D). To investigate whether ERK1/2 activation could be responsible for the lower cytotoxic effect of DMF against the BC3 cell line, we pre-treated these cells with PD98059 ERK1/2 inhibitor before exposing them to DMF and found that such a combination was more cytotoxic than DMF or PD98059 alone (Figure 3E). The efficiency of ERK1/2 inhibition by PD98059 was also demonstrated by western blot analysis, and the higher cytotoxicity of DMF in combination with PD98059 was confirmed by the increase in PARP cleavage (Figure 3F).

### 2.4. The Induction of a Pro-Survival Autophagy by ERK1/2 Activation Counteracts the Cytotoxic Effect of DMF in BC3 Cell Line

**Figure 4:**
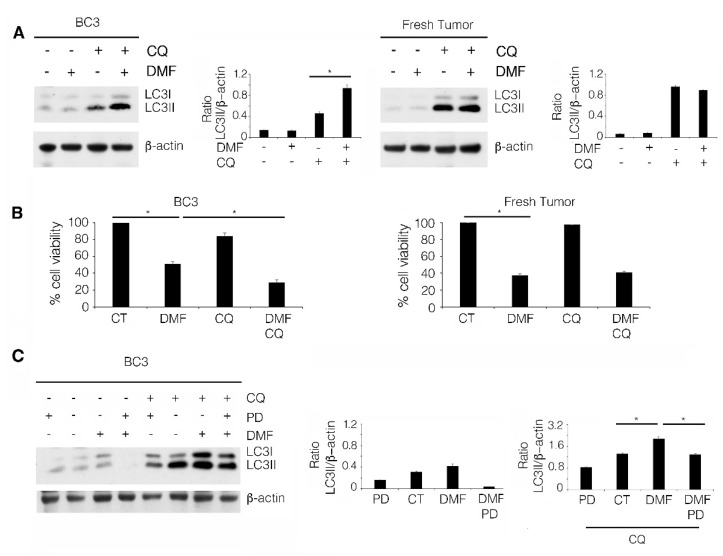
ERK1/2 activation triggers a pro-survival autophagy, reducing the DMF cytotoxicity in the BC3 cell line. (**A**) Western blot analysis to detect LC3II levels in BC3 cells and PEL fresh tumors, treated or not (CT) with 50 µM DMF for 24 h, in the presence of 20 µM chloroquine (CQ). β-actin was used as loading control, and one representative experiment out of three is shown. The histograms represent the mean plus SD of the densitometric analysis of the LC3II/β-actin ratio of three different experiments. (**B**) Percentage of cell viability of BC3 cells and PEL fresh tumors, treated or not (CT) with 50 µM DMF for 24 h, in the presence or absence of 20 µM CQ. (**C**) Western blot analysis to detect LC3II levels in BC3 cells, treated or not (CT) with 50 mM DMF for 24 h, in the presence or absence of 20 µM chloroquine (CQ) and PD98059 EK1/2 (PD) inhibitor. β-actin was used as loading control, and one representative experiment out of three is shown. The histograms represent the mean plus SD of the densitometric analysis of the LC3II/β-actin ratio of three different experiments. In the figure, mean ± standard deviation (SD) of three independent experiments is shown. * *p* < 0.05.

We then evaluated the impact of DMF on autophagy, in the BC3 cell line and in fresh PEL cells, to investigate whether a different activation of this process could contribute to the different cytotoxic effect of DMF against these two cell types. To this end, considering that p62/SQSTM1 was transcriptionally upregulated by DMF through NRF2 activation (Figure 1C,D), we decided that p62/SQSTM1 could not be used as an autophagic marker; thus, we assessed the expression level of LC3II, the lipidated form of LC3 that is formed during autophagy activation. LC3I/II was evaluated in the presence or in the absence of chloroquine, as this drug prevents LC3II degradation, which also occurs during the autophagic process. We found that LC3II increased in the presence of chloroquine in the BC3 cell line, while such increase was not observed in fresh PEL cells, suggesting that autophagy was only activated in the BC3 cell line (Figure 4A). We then observed that chloroquine increased the cytotoxic effect of DMF in the BC3 cell line but not in fresh PEL cells (Figure 4B), indicating that autophagy played a pro-survival role in the DMF-treated BC3 cell line. We finally investigated whether the induction of autophagy could correlate with ERK1/2 activation in the DMF-treated BC3 cell line. To this end, we pre-treated these cells with PD98059 ERK1/2 inhibitor and found that it counteracted autophagy induction by DMF (Figure 4C). Overall, these findings suggest that DMF, by inducing ERK1/2 activation, triggered autophagy in the BC3 cell line, and this effect reduced its cytotoxic effect.

## 3. Discussion

This study shows for the first time that DMF could represent a promising therapeutic option against PEL, particularly against fresh PEL cells, in which such treatment did not trigger ERK1/2 and autophagy activation, as a defense mechanism that counteracted its cytotoxic effect. Besides ERK1/2, which was hyper-phosphorylated by DMF at the dose of 50 µM in the BC3 cell line, the mTOR/p-4EBP1 axis was activated by DMF in both PEL cell lines and fresh PEL cells when it was used at the lower dose of 20 µM. This pathway also reduced DMF cytotoxicity, as suggested by the stronger cell death induced by the combination of DMF with NVP-BEZ235. Drug resistance is a major obstacle to cancer therapies; therefore, it is of pivotal importance to unveil the molecular mechanisms and the processes that sustain cancer cell survival in the course of anti-cancer treatments, such as p-4EBP1 and ERK1/2 in the case of PEL cells treated by DMF at low and high doses, respectively. However, despite this resistance, DMF still induced a cytotoxic effect against PEL cells, and this occurred in correlation with NRF2 activation, ROS reduction, and de-phosphorylation of STAT3. These lymphoma cells are indeed strongly addicted to this pathway [15], which is constitutively activated by the release of pro-inflammatory cytokines that act in an autocrine fashion and, due to the expression of proteins encoded by the oncovirus KSHV [31], are carried in a latent state in PEL cells. Of note, STAT3 inhibition by DMF correlated with the reduction of IL-6 and IL-10, in accordance with previous findings [32], and these pro-inflammatory and immunosuppressive cytokines are known to be strongly involved in PEL cell survival [24]. Of note, it has been reported that one of the most important therapeutic effects of DMF relies on its anti-inflammatory properties, which is why this drug has been registered for the treatment of inflammatory-based diseases such as psoriasis and multiple sclerosis [33,34]. Regarding cancer, previous studies have demonstrated the anti-cancer effects of DMF against several solid cancers [35,36] and hematologic cancers, including cutaneous T-cell lymphoma [37], diffuse large B cell lymphoma (DLBL), and HTLV-1-associated adult T cell leukemia [38]. At the molecular level, NF-kB was found to be inhibited by DMF and, in the latter two cancer types, the de-phosphorylation of STAT3 was also induced. The other important effect of DMF that we observed in this study was that it promoted autophagy in the BC3 cell line, while this process was not activated in fresh PEL cells. Autophagy represented a pro-survival mechanism induced in correlation with the activation of the ERK1/2 pathway [39]; indeed, through autophagy, cancer cells may often be resistant to chemo and radiotherapies. Therefore, it is worthwhile to evaluate the impact of treatments on this process and to explore whether its inhibition can be used as strategy to potentiate the outcome of treatments [27]. Interestingly, autophagy was not induced by DMF in fresh PEL cells; indeed, we observed a higher cytotoxic effect against these cells.

## 4. Materials and Methods

### 4.1. Cell Culture and Reagents

BC3 (ATCC, CRL-2277) and BCBL1 (kindly provided by Prof. P. Monini, National AIDS Center, Istituto Superiore di Sanità, Rome, Italy) are human B cell lines derived from Primary Effusion Lymphoma (PEL). Fresh PEL cells were obtained from ascites grown in SCID mice. Mice were engrafted with the BC3 cell line; after 30/35, when mice became ill, they were sacrificed, and ascites were withdrawn. Cells were cultured in RPMI 1640 (Sigma, R0883) containing 10% fetal calf serum (Euroclone, ECLS0180L), L-glutamine (2 mM), streptomycin (100 µg/mL), and penicillin (100 U/mL) (Gibco, 10378-016) at 37 °C in 5% CO2. Cells were treated with the following chemicals: Dimethyl fumarate (DMF) (20 and 50 µM; Sigma-Aldrich, St. Louis, MO, USA, cat. n. 242926), Chloroquine (CQ) (20 µM; Sigma-Aldrich, cat. n. C6628), NVP-BEZ235 (BEZ) (dual PI3K/mTOR inhibitor; 100 nM; Selleckchem, Planegg, Germany, cat. n. S1009), Brusatol (BRU) (NRF2 inhibitor; 5 nM; Sigma-Aldrich, St. Louis, MO, USA, cat. n. SML1868), PD98059 (PD) (ERK inhibitor; 20 µM; Sigma-Aldrich, St. Louis, MO, USA, P215).

Cells were treated with DMF for 24 h, Chloroquine was added for the last 18 h, and all inhibitors were added to the cells 30 min before DMF.

### 4.2. Trypan Blue Exclusion Assay

Cells were harvested after treatments and then centrifuged and counted by Trypan blue exclusion assay (Trypan blue, Sigma-Aldrich, St. Louis, MO, USA, cat. n. T8154) by using a Neubauer hemocytometer in a phase contrast microscope. Each experiment was performed in triplicate.

### 4.3. Western Blot Analysis

Cells were harvested and centrifuged, and the resulting pellets were lysed in modified RIPA buffer (150 mM NaCl, 1% NP40, 50 mM Tris–HCl pH 8, 0.5% deoxycholic acid, 0.1% SDS, 1% Triton X-100 protease and phosphatase inhibitors). Protein concentration was determined by using a BCA protein assay kit (Sigma-Aldrich, St. Louis, MO, USA, cat. n. 71285-M) and, finally, 10 μg of each lysate was loaded on 4–12% NuPage Bis Tris gels (Thermo Fisher, Waltham, MA, USA, NP0323). After electrophoresis, proteins were blotted on Nitrocellulose membrane (Whatman, GE Healthcare, Chicago, IL, USA 10401196). Membranes were treated in PBS 0.2% Tween-20 (PBS/T) containing BSA 3% for 30 min at RT and then incubated with primary antibody overnight at 4 °C. After 3 washes with PBS containing 0.2% Tween-20, membranes were probed with suitable HRP-conjugated secondary antibodies (Santa Cruz Biotechnologies, Dallas, TX, USA,) and finally subjected to ECL reaction with specific substrate (Advansta, San Jose, CA, USA, 12045-D20).

### 4.4. Antibodies

The following antibodies were diluted in PBS/T containing BSA 3% and used to detect the expression of specific proteins on western blot membranes:

- mouse monoclonal anti-NQO1 (1:500) (Santa Cruz Biotechnologies, Dallas, TX, USA, cat. n. sc-32793),
- rabbit polyclonal anti-phospho-4E-BP1 (Thr37/46) (1:200) (Cell Signaling, Danvers, MA, USA, cat. n. 2855),
- rabbit polyclonal anti-4E-BP1 (1:200) (Cell Signaling, Danvers, MA, USA, cat. n. 9452),
- rabbit polyclonal anti-PARP1(1:1000) (Proteintech, Manchester, UK, cat. n. 13371-1),
- rabbit polyclonal anti-phospho STAT3 Tyr705 (1:500) (Santa Cruz Biotechnologies, Dallas, TX, USA, cat. n. sc-8059),
- mouse monoclonal anti-STAT3 (1:100) (BD Transduction Lab, Franklin Lakes, NJ, USA, cat. n. 610189),
- rabbit polyclonal anti-LC3I/II (1:1000) (Novus, Littleton, CO, USA, cat. n. NB100-2220),
- mouse monoclonal anti-p62/SQSTM1 (1:300) (BD Transduction Lab, Franklin Lakes, NJ, USA, cat. n. 610832),
- rabbit polyclonal anti-ERK1 (1:200) (Santa Cruz Biotechnologies, Dallas, TX, USA, cat. n. sc-93),
- rabbit polyclonal anti-ERK2 (1:200) (Santa Cruz Biotechnologies, Dallas, TX, USA, cat. n. sc-154),
- mouse monoclonal anti-p-ERK (1:500) (Santa Cruz Biotechnologies, Dallas, TX, USA, cat. n. sc-7383),
- mouse monoclonal anti-p-ERK (1:500) (Santa Cruz Biotechnologies, Dallas, TX, USA, cat. n. sc-7383),
- mouse monoclonal anti-MFN2 (1:200) (Santa Cruz Biotechnologies, Dallas, TX, USA, cat. n. sc-515647).
- mouse monoclonal anti-β-actin (1:10,000) (Sigma Aldrich, St. Louis, MO, USA #A5441) was used as loading control.

Goat anti-rabbit IgG- horseradish peroxidase HRP (Santa Cruz Biotechnologies, Dallas, TX, USA, sc-2004) and goat anti-mouse IgG-horseradish peroxidase HRP (Santa Cruz Biotechnologies, Dallas, TX, USA, sc-2005) were diluted 1:10,000 in PBS/T containing BSA 3% and used as secondary antibodies.

### 4.5. Densitometric Analysis

Bands evidenced by western blot were subjected to densitometric analysis by using ImageJ software, which was downloaded from the NIH web site (available online: http://imagej.nih.gov (accessed on 5 May 2022)).

### 4.6. Quantitative Real-Time PCR

Total RNA levels from PEL cell lines treated with DMF (50 μM) for 24 h and from those untreated (CT) were extracted by using TRIzol™ Reagent (Thermo Fisher, Waltham, MA, USA, cat. n.15596026) according to the manufacturer’s instructions. After in vitro reverse transcription, quantitative Real-Time PCR (qRT-PCR) was performed and TaqMan gene expression assay specific to p62 mRNA was used as described elsewhere [40]. All values were normalized to β-actin (endogenous gene controls).

### 4.7. Measurement of Intracellular Reactive Oxygen Species (ROS) Production

Cells were treated with 10 µM 2′,7′-dichlorofluorescein diacetate (DCF-DA; Molecular Probes, CA, USA) for 20 min at 37 °C, washed with 1x PBS, and then analyzed in FL-1 by a FACS-Calibur flow cytometer (BD, Biosciences, as previously described) [41]

### 4.8. Chemiluminescence Immunometric Assay

Cells were harvested 24 h after DMF treatments and then centrifuged. Supernatants were collected to measure Interleukin-10 (IL-10) and Interleukin-6 (IL-6) by Magnetic Luminex assay (R&D systems Bio-Techne Abingdon, UK, LXSAHM) according to the manufacturer’s instructions.

### 4.9. Immunofluorescence Assay (IFA)

Fresh tumors cells were washed with PBS and then layered onto multispot microscope slides and air dried. Cells were fixed with 2% paraformaldehyde (Electron Microscopy Science, Hatfield, PA, USA) for 30 min and permeabilized with 0.1% Triton X-100 (Sigma-Aldrich, St. Louis, MO, USA) for 5 min. After 3 washes and 30 min incubation with 1% glycine 3% BSA, cells were incubated with the primary antibody (anti-LANA, MBL cat. n. D325-3, 1:500) for 1 h at RT. After 3 washes with PBS, slides were subjected to secondary antibody incubation (Sheep anti-mouse IgG Cy-3, Jackson, 1:1500) for 30 min at RT, washed, stained with DAPI (Sigma Aldrich, St. Louis, MO, USA), and finally analyzed with a Olympus BX53 fluorescence microscope [42].

### 4.10. Statistical Analysis

The results are represented by the mean ± standard deviation (S.D.) of at least three independent experiments, and a two-tailed Student’s t-test was used to demonstrate statistical significance. Differences were considered statistically significant when the *p*-value was at least <0.05.

## 5. Conclusions

In conclusion, this study presents for the first time that DMF could be a promising treatment against PEL, particularly because it was even more effective against fresh lymphoma cells. We also evidenced the molecular pathways underlying its cytotoxic effect and those that counteracted it, which could be targeted to potentiate the outcome of DMF treatment against PEL, an aggressive lymphoma that still remains an orphan drug cancer.

## Data Availability

The datasets generated and analyzed during the current study are available from the corresponding author upon reasonable request.
